# Modeling, analysis, and experimental validation of a swash plate compressor for automotive air conditioning

**DOI:** 10.1038/s41598-025-27318-w

**Published:** 2025-11-29

**Authors:** R. K. Mohammed, Hany A. Mohamed, MR. Abdelaal

**Affiliations:** 1 Manufacturing Department, Modern Academy for Engineering and Technology, Cairo, 11571 Egypt; 2 Department of Mechanical Power Engineering, Faculty of Engineering, Assiut University, Asyut, 71516 Egypt

**Keywords:** Air conditioning system, Swash plate compressor, Kinematic and dynamic analysis, Swash plate inclination angle, Engineering, Electrical and electronic engineering, Mechanical engineering

## Abstract

This paper presents a comprehensive study of a swash plate compressor, integrating both theoretical modeling and experimental validation. A mathematical model is developed to precisely describe the swash plate’s motion and to derive the average power input to the compressor. This derivation considers the compressor’s geometric parameters and angular speed under various operating conditions. Complementary to the theoretical analysis, an experimental investigation of an automotive air conditioning system equipped with a swash plate compressor was conducted. Both theoretical and experimental findings consistently demonstrate that the average power consumed by the swash plate compressor is primarily dependent on the swash plate inclination angle, rotational speed, and system pressure. A key conclusion drawn from this research is the critical importance of a small swash plate inclination angle. Such an angle is shown to be essential for minimizing power losses attributed to friction between the slipper and the swash plate, thereby reducing the overall shaft power required by the compressor. Furthermore, for design scenarios demanding both a long stroke and minimal shaft power for the swash plate compressor, the present analysis provides a crucial framework for selecting the optimal inclination angle and stroke length. Experimental results indicate that achieving high coefficients of performance (COP) and volumetric efficiencies necessitates low rotational speeds, high cooling capacities, and a reduced shaft power per unit mass flow rate of refrigerant. The coefficient of performance relative to the corresponding Carnot cycle is observed to decrease hyperbolically with an increase in the shaft power per unit mass flow rate of refrigerant. Importantly, the calculated shaft power values from the theoretical model provides a reasonable approximations agreement with those obtained from a simulation program developed by the compressor’s manufacturer [8] across various operating conditions.

## Introduction

Air conditioning has become an increasingly vital component in recent years, particularly in regions with hot climates. Automotive air conditioning systems represent a significant advancement in this field, especially for compact vehicles. Among the various compressor types, swash plate compressors are widely favored in automotive air conditioning systems due to their numerous advantages. These include their relatively low cost, ready availability, compact design, reliable operation across a broad range of mass flow rates, adaptability to varying engine speeds, and the incorporation of a rapid idle runner.

The swash plate mechanism serves as a fundamental kinematic linkage that converts rotary motion into linear reciprocating motion and vice versa. In this configuration, pistons are connected to an inclined swash plate through spherical joints. Previous research, such as that by David Edgar Evans^1^, has explored the kinematics of the swash plate mechanism using dual-number transformation matrices. However, his study primarily focused on the displacements of each joint within the mechanism and did not delve into the velocity or dynamic analysis of the swash plate mechanism.

Further investigations^2–4^ have examined the variation of cylinder pressure throughout a complete pump cycle, including the effects of different valve plate designs on this pressure variation. The modeling and design of an axial piston pump with variable geometric displacement were detailed in^3^. The cylinder pressure and the torque acting on the swash plate were computed in 4 for two distinct valve plate types. The calculated values demonstrated good agreement with measured values when using a valve plate with wide, short, and deep slots.

A mathematical model for axial piston swash plate pumps featuring conical cylinder blocks was presented in^5^. The findings from this study indicated that both the torque acting on the swash plate in the direction perpendicular to its inclination and the torque acting on the drive shaft remain nearly constant under specific operating conditions. Furthermore, these torques were observed to increase linearly with increasing delivery pressure and/or increasing swash plate inclination angle. A model for a swash plate type hydraulic motor was developed in^6^, incorporating piston dynamics and the effects of port plate geometry. Low-speed characteristics were analyzed in both the time domain and phase plane for stability and the type of limit cycle behavior.

More recently, a novel pump design incorporating a piston-bore spring was implemented^7^. This piston-bore spring was integrated into the design to stabilize the cylinder block against the valve plate and to force the pistons in a negative direction. A general equation describing the motion of the swash plate for this innovative design was derived. Recent studies have continued to explore the optimization and performance analysis of swash plate compressors. For instance, researchers have focused on improving energy efficiency and reducing the size of these compressors for electric vehicles^11^. Advanced control strategies have also been investigated to enhance the performance of swash plate compressors under varying operating conditions^12^. Furthermore, computational fluid dynamics (CFD) simulations have been employed to analyze the flow characteristics and pressure distribution within the compressor, aiding in the design of more efficient and reliable systems^13^. Studies also focus on alternative refrigerants to improve system performance and address environmental concerns^14,15^. These efforts highlight ongoing research and development aimed at advancing the design and performance of swash plate compressors in automotive air conditioning systems.

The present work aims to provide a comprehensive description of the swash plate mechanism within a typical automotive air conditioning system. A theoretical kinematic and dynamic analysis of the swash plate compressor is conducted. The derived equations describe the displacements and velocity of the swash plate mechanism. The average values of shaft torque and shaft power for the swash plate compressor can be expressed as functions of the piston’s angular position, the swash plate inclination angle, the eccentricity of the piston’s center from the swash plate’s center, the pressure-holding angle, and the suction and discharge pressures of the compressor. Concurrently, the performance of an automotive air conditioning system, including a swash plate type compressor, is investigated experimentally. The results from the experimental and theoretical analyses are then compared with those obtained from a simulation program developed by the manufacturer of the swash plate compressor used in this study (Sanden International (Europe) Ltd) 8.

### Description of swash plate compressor model

**Fig. 1:**
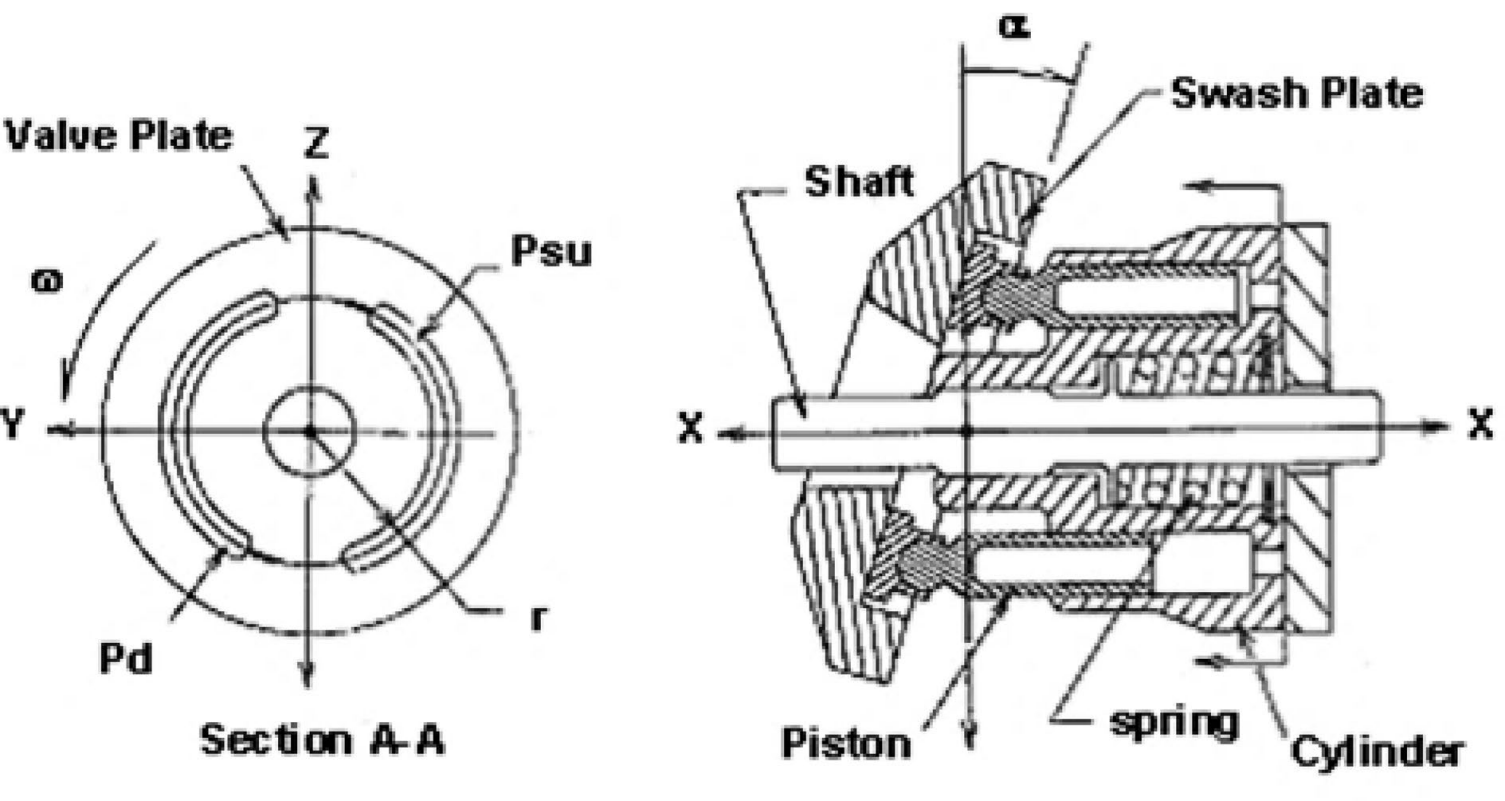
Swash plate compressor.

Figure 1 illustrates a typical cross-section of a swash plate compressor. The port plate, or valve plate, is positioned on the left side of the figure. The compressor consists of multiple pistons housed within a cylinder block. These pistons are arranged at equal intervals in a circular array around the cylinder axis. The cylinder block is rigidly held against the port plate. A thin film of oil separates the cylinder block and the port plate, forming a hydrodynamic bearing surface between the two components. The pistons extend from the left end of the cylinder block and terminate in a ball joint with slippers. These slippers are held against the face of the swash plate by a pull plate, retainer, and spring. An actuator (not shown) is employed to control the swash plate angle, α.

During the rotation of the swash plate, each piston periodically passes over both the discharge and suction ports. As the pistons pass over the suction port, the piston retracts from the cylinder, allowing gas to enter the piston cylinder. Conversely, when the pistons pass over the discharge port, the piston advances into the cylinder block, expelling the gas from the piston cylinder. This reciprocating motion is repeated for each cycle, fulfilling the fundamental task of gas compression. By varying the swash plate angle α, the piston stroke length is adjusted, which in turn modifies the torque required for compression.

### Theoretical work

#### Assumptions

• Leakage effects were not considered.

- Viscous damping effects were neglected.
- Compressibility and pressure dynamics effects were disregarded.
- Friction at the valve plate interface and piston-bore friction were not considered.
- The increase in normal reaction due to cantilever piston effects was assumed to be absorbed within the model’s structure.
- Hydrodynamic balance effects between the slippers and the swash plate, and between the valve plate and piston cylinder, were ignored.
- Swash plate dynamics were integrated into the reaction force between the swash plate and the slippers.
- Clearances and gaps at the piston interface and ball/slipper interface were neglected.
- Shaft stiffness was disregarded.
- A lumped representation of the valve plate timing was utilized.
- The swash plate angle was assumed to be constant.
- The suction and delivery ports of the port plate were assumed to be identical.

### Kinematics analysis

**Fig. 2:**
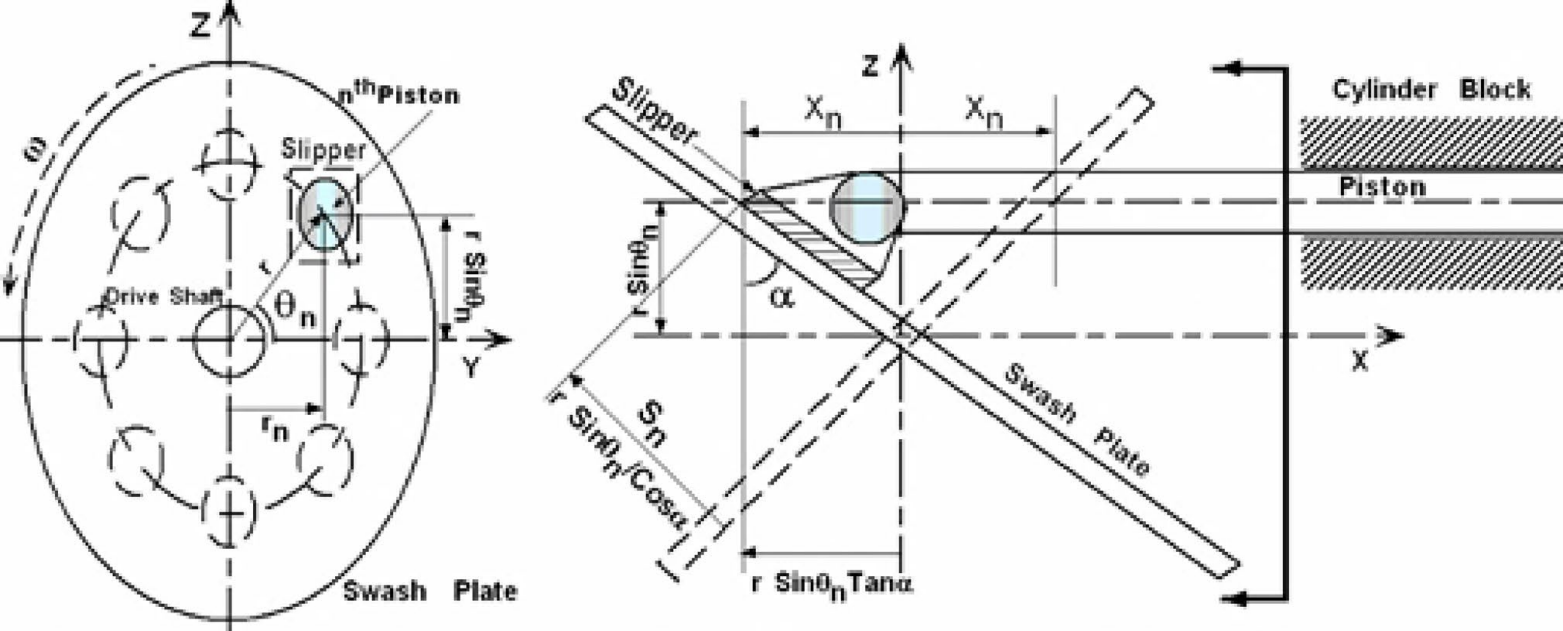
Schematics drawing for the swash plate compressor mechanism.

Figure 2 illustrates the compressor mechanism under investigation. The right side of the figure depicts the motions of the piston and slipper relative to the swash plate. The left side of the figure, conversely, shows the motion of the slipper relative to the swash plate. It can be observed that the displacement of the X_n_ of the piston as function of its angular position θ_n_, the swash plate inclination angle α and the eccentricity of the piston’s center from the swash plate’s center **r** is given by:

1 $$\:{X}_{n}=r\left(Tan\alpha\:\right)\left(Sin{\theta\:}_{n}\right)$$

Thus, half of the stroke length L which equals the maximum value of X_n_, becomes (${\rm L/2=r\:Tan_{\alpha}}$). The position of the slipper on the swash plate plane normal and along Y axis (s_n_, r_n_) in terms of r, α and θ can be also obtained as:

2 $$\:{r}_{n}=r\left(Cos{\theta\:}_{n}\right)$$

3 $$\:{S}_{n}=\frac{r\left(Sin{\theta\:}_{n}\right)}{\left(Cos\alpha\:\right)}$$

The angle of the n^th^ piston θ_n_ in terms position the angle of the first piston θ_1_ was considered as:

4 $$\:{\theta\:}_{n}={\theta\:}_{1}+\frac{2\pi\:\left(n-1\right)}{Np}$$

Where n is the piston number and Np is the number of the pistons.

### Dynamics analysis

**Fig. 3:**
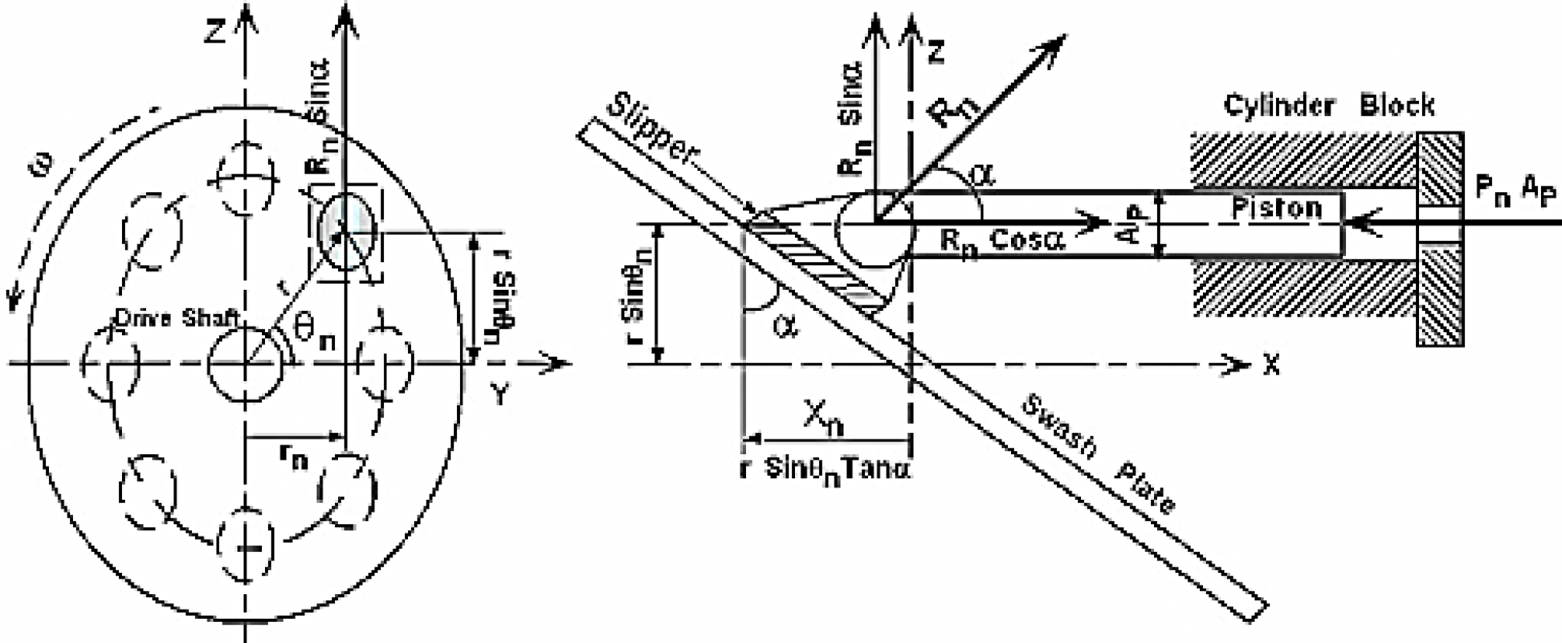
Forces acting on the cylinder from a single piston.

Figure 3 presents a schematic diagram of a single piston within the cylinder during compressor operation, illustrating the forces acting on the cylinder from that single piston. From the left side of this figure, it can be deduced that the torque acting about the drive shaft center by a single piston is:

5 $$\:{T}_{n}=\left({R}_{n}{sin}\alpha\:\right){r}_{n}$$

Where ${\rm R_{n}\:sin\:\alpha}$ equals the vertical component of the swash plate force acting on the piston and r_n_ is the horizontal distance of the n^th^ piston away from the Z-axis. Summing the forces in the X-direction for a single piston and setting them equal to the piston’s inertia, it can be obtained that

6 $$\:{R}_{n}=\frac{{m}_{p}{\ddot{X}}_{n}+{P}_{n}{A}_{p}}{\left(Cos\alpha\:\right)}$$

Where m_P_ is the mass of a single piston, P_n_ is the pressure acting on the n^th^ piston and A_P_ is the cross-sectional area of a single piston. The instantaneous translational acceleration $\:{\ddot{X}}_{n}$ is obtained by differentiating Eq. (1) twice with respect to time as:

7 $$\:{\ddot{X}}_{n}=-r{\omega\:}^{2}\left(Tan\alpha\:\right)\left(Sin{\theta\:}_{n}\right)$$

Where ω is the angular velocity of the swash plate (i.e. the angular velocity of the driven shaft). Using Eqs. (2), (5), (6) and (7) the net instantaneous torque T exerted on the driven shaft may be expressed as

8 $$\:T={\sum\:}_{n=1}^{Np}rCos{\theta\:}_{n}Tan\alpha\:\left({P}_{n}{A}_{p}-r{\omega\:}^{2}{m}_{p}Tan\alpha\:Sin{\theta\:}_{n}\right)$$

By replacing the summation sign in Eq. (8) with an integral representation, the average torque Tav exerted on the driven shaft is given by

9 $$\:{T}_{av}=\frac{Np}{2\pi\:}{\int\:}_{0}^{2\pi\:}rCos{\theta\:}_{n}Tan\alpha\:\left({P}_{n}{A}_{p}-r{\omega\:}^{2}{m}_{p}Tan\alpha\:Sin{\theta\:}_{n}\right)d{\theta\:}_{n}$$

Then the theoretical power input to the shaft can be calculated as

10 $$\:SP={T}_{av}\omega\:$$

**Fig. 4:**
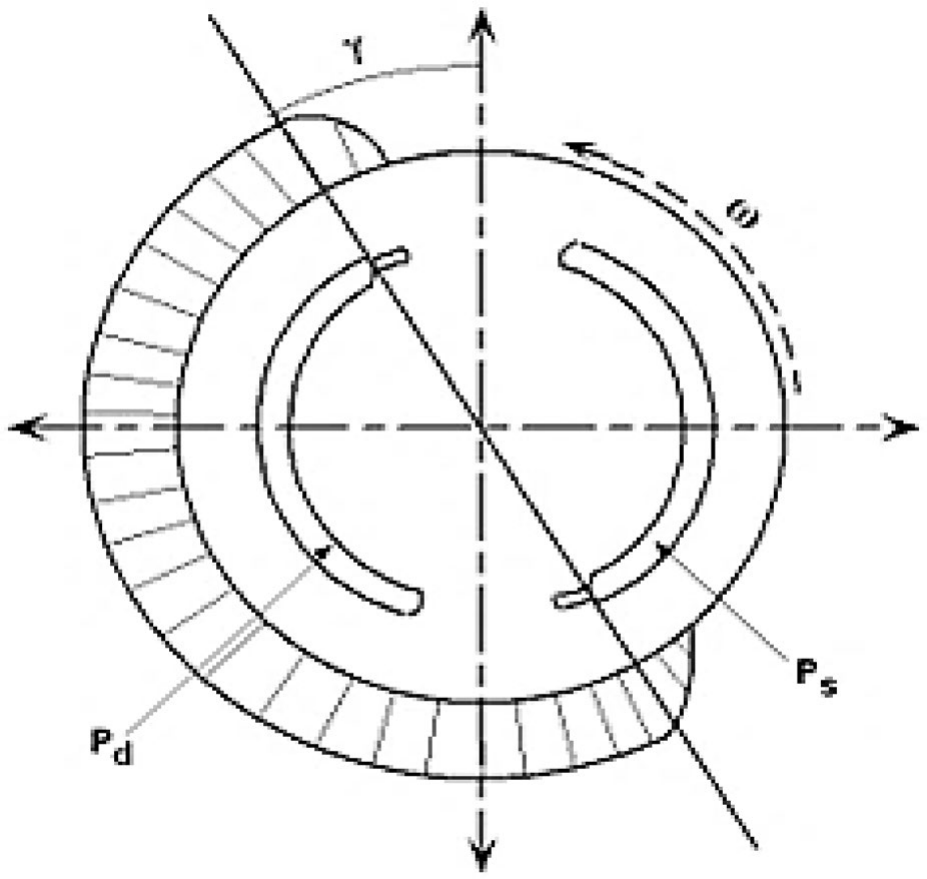
Approximate pressure profile schematic with the magnitude of the discharge pressure P_d_ referenced from the magnitude of the suction pressure P_s_.

Figure 4 represents schematically the approximate pressure profile P_n_ of the n^th^ piston as it is popular in industry^2^. This schematic appears that the piston accompanies a constant pressure as it passes directly over either port (suction or discharge port) and that it undergoes a transition in pressure as it passes over the slots on the valve plate. This transition occurs through some average angular distance γ which is commonly referred to as the pressure carry over angle. It is assumed that the pressure transition between ports occurs linearly over the range of the pressure carried over angle γ as approximated in^2^ as

11 $$\:{P}_{n}=\left\{\begin{array}{c}{P}_{d}\:\:\:\:\:\:\:\:\:\:\:\:\:\:\:\:\:\:\:\:\:\:\:\:\:\:\:\:\:\:\:\:\:\:\:\:\:\:\:\:\:\:\:\:\:\:\:\:\:\:\:\:\:\:\:\:\:\:\:\:\:\:\:\left(3\pi\:/2+\gamma\:\right)<{\theta\:}_{n}<\pi\:/2\\\:{P}_{d}-\left({P}_{d}-{P}_{s}\right)\left({\theta\:}_{n}-\pi\:/2\right)/\gamma\:\:\:\:\:\:\:\:\:\:\:\:\:\:\:\:\:\:\:\:\:\:\:\:\:\:\:\pi\:/2<{\theta\:}_{n}<\left(\pi\:/2+\gamma\:\right)\\\:{P}_{s\:\:\:\:\:\:\:\:\:\:\:\:\:\:\:\:\:\:\:\:\:\:\:\:\:\:\:\:\:\:\:\:\:\:\:\:\:\:\:\:\:\:\:\:\:\:\:\:\:\:\:\:\:\:\:\:\:\:\:\:\:\:\:\:\:\:\:\:\:\:\:\:\:\:\:\:\:\:\:\:\:\:\:\:\:\:\:\:\:\:\:\:\:\:}\left(\pi\:/2+\gamma\:\right)<{\theta\:}_{n}<3\pi\:/2\\\:{P}_{s}+\left({P}_{d}-{P}_{s}\right)\left({\theta\:}_{n}-3\pi\:/2\right)/\gamma\:\:\:\:\:\:\:\:\:\:\:\:\:\:3\pi\:/2<{\theta\:}_{n}<{\theta\:}_{n}<\left(3\pi\:/2+\gamma\:\right)\end{array}\right.$$

According to the assumption used in the above analysis, the value of the average torque obtained from Eq. (9) represents the minimum torque input to the driven shaft for achieving P_n_ pressure corresponding to certain values of delivery and suction pressures (P_d_ and P_s_). A value of **γ = 15**^°^ was assumed, which is a typical value for such compressors, reflecting the transition zone between the suction and discharge ports on the valve plate. Consequently, the theoretical shaft power given by Eq. (10) represent approximately the minimum shaft power.

### Experimental test rig and procedure

**Fig. 5:**
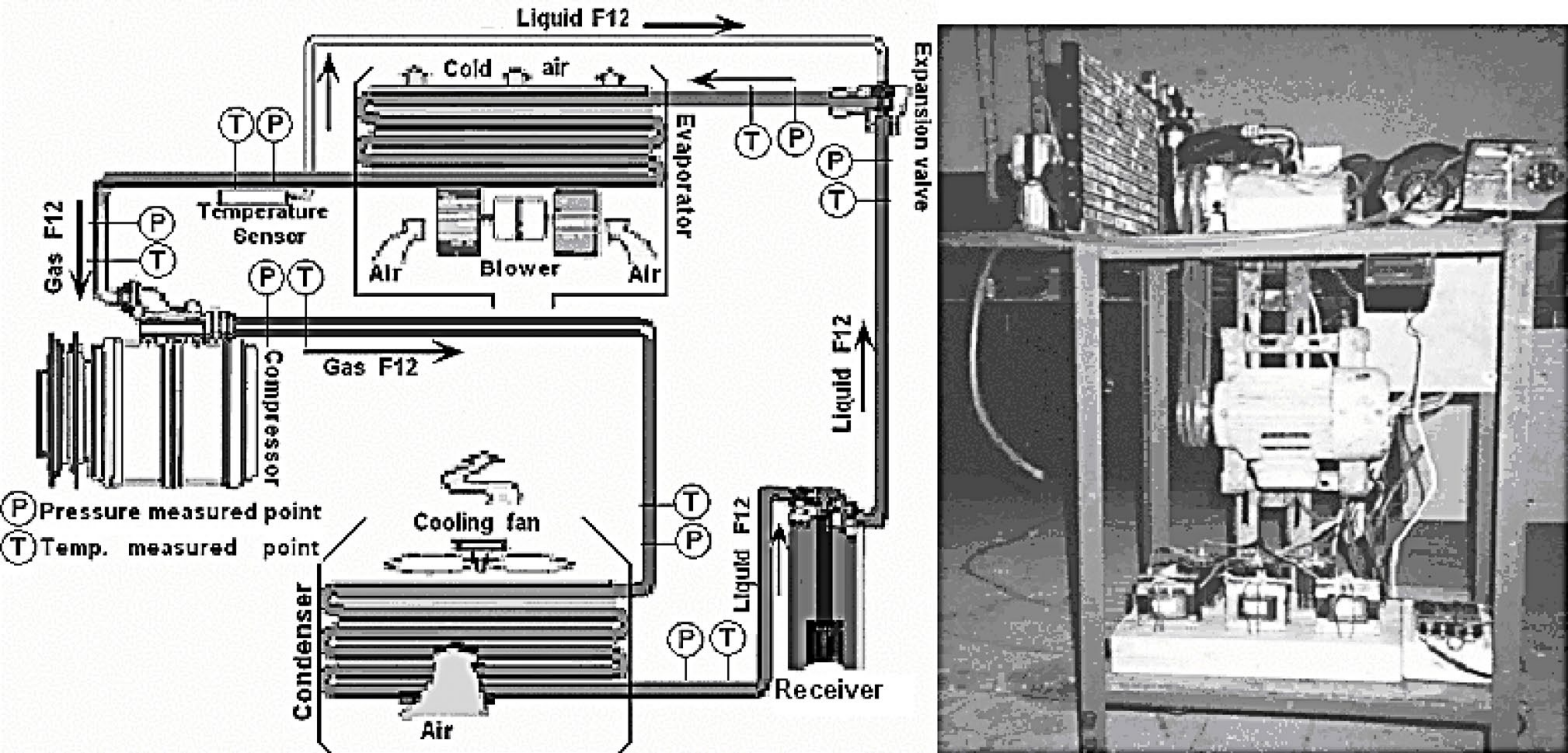
Schematic drawing and photographic picture.

A schematic drawing and photographic picture for the experimental test rig used in the present work are shown in Fig. 5. The experimental test rig is like car air conditioning, and it is composed mainly from evaporator, swash plate compressor, condenser and expansion valve. The compressor discharges high temperature and high-pressure refrigerant (F12) that contains the heat absorbed from the evaporator and the heat created by the compressor in a discharge stroke. This gaseous refrigerant flows into the condenser. In the condenser, the gaseous refrigerant is condensed into liquid refrigerant. The liquid refrigerant then flows into the receiver, which stores and filters the liquid refrigerant until the evaporator requires it. The expansion valve changes the liquid refrigerant into a low temperature, low pressure liquid and gas mixture. This cold refrigerant flows to the evaporator. Vaporizing the liquid in the evaporator takes heat from the warm air stream passing through the evaporator. All the liquid will change into a gaseous refrigerant in the evaporator and only gaseous refrigerant, which has a latent heat, goes into the compressor. The process is then repeated. The swash plate compressor (Sanden SD-508 type) is driven by a constant speed AC electric motor attached with the compressor through a belt and magnetic clutch. The specifications of the swash plate compressor are given in Table 1. A wattmeter was used to measure the electric power supplied to the motor. The shaft power input to the compressor SP was assumed 90% of the motor power. The speed of the compressor shaft was measured by a tachometer with uncertainties of ± 50 rpm. The pressure and temperature of the refrigerant were measured at the locations shown in Fig. 5 with uncertainties of ± 10 kPa and ± 0.2 °C. The pressure and the temperature of the air were also measured at various positions. The velocities of the air flowing V_a_ through the ducts surrounded the evaporator and the condenser were measured by using air anemometer with uncertainties of ± 0.5 m/s. The pressure and the temperature of the air were also measured at entrance and departure the ducts on various points with uncertainties of ± 10 kPa and ± 0.5 °C. The experimental tests were run at constant speeds of 1690, 1290 and 1140 rpm for varying the air flowing V_a_ through the ducts. Sixty data points were recorded through the test. At the beginning of each experiment, the equipment was operated under the presumed test conditions for fifteen minutes before readings were recorded to ensure steady state condition. The mass flow rate of the air flowing through the ducts m_a_ was calculated as follow:

Figure 5 presents both a schematic diagram and a photograph of the experimental test rig employed in the current study. The experimental setup simulates an automotive air conditioning system, primarily consisting of an evaporator, a swash plate compressor, a condenser, and an expansion valve.

The compressor discharges high-temperature, high-pressure refrigerant (F12) that contains heat absorbed from the evaporator and heat generated by the compressor during the discharge stroke. This gaseous refrigerant then flows into the condenser. Within the condenser, the gaseous refrigerant is condensed into liquid refrigerant. The liquid refrigerant subsequently flows into the receiver, which stores and filters the liquid refrigerant until it is required by the evaporator. The expansion valve converts the liquid refrigerant into a low-temperature, low-pressure liquid-gas mixture. This cold refrigerant then flows into the evaporator. The vaporization of the liquid in the evaporator absorbs heat from the warm air stream passing through it. All the liquid refrigerants will transform into gaseous refrigerant in the evaporator, ensuring that only gaseous refrigerant, containing latent heat, enters the compressor. The entire refrigeration cycle then repeats.

The swash plate compressor (Sanden SD-508 type) is driven by a constant-speed AC electric motor connected to the compressor via a belt and an electromagnetic clutch. The specifications of the swash plate compressor are provided in Table 1. A wattmeter was used to measure the electrical power supplied to the motor. The shaft power input to the compressor (SP) was assumed to be 90% of the motor power. The compressor shaft speed was measured by a tachometer with an uncertainty of ± 50 rpm. Refrigerant pressure and temperature at the locations indicated in Fig. 5 were measured with uncertainties of ± 10 kPa and ± 0.2 °C, respectively. Air pressure and temperature were also measured at various locations. Air flow velocities Va through the ducts surrounding the evaporator and condenser were measured using an anemometer with an uncertainty of ± 0.5 m/s. Air pressure and temperature were also measured at the inlet and outlet of the ducts at different points with uncertainties of ± 10 kPa and ± 0.5 °C.

Experiments were conducted at constant compressor speeds of 1690, 1290, and 1140 rpm for varying air flow velocities Va through the ducts. Sixty data points were recorded during the testing. At the beginning of each experiment, the apparatus was operated under the assumed test conditions for fifteen minutes prior to recording readings to ensure a steady state. The mass flow rate of air flowing through the ducts, m_a_ was calculated as:

12 $$\:{m}_{a}=\frac{{P}_{a}{V}_{a}{A}_{d}}{R{T}_{a}}$$

where P_a_, T_a_, V_a_, R and A_d_ are the pressure, the average temperature and the average velocity at air duct entrance, gas constant for air and the air duct cross sectional area respectively. The mass flow rate of the refrigerant m_r_ is calculated from heat balance between heat added or rejected from the evaporator or the condenser with heat transfer from or to the air flowing through the duct by using Eq. (13). Only when the heat losses were within 5%, were the results accepted.

13 $$\:{m}_{r}=\frac{{m}_{a}C{p}_{a}\varDelta\:{T}_{a}}{dh}$$

where ΔT_a_ equals the difference between the average temperatures of the air at entrance and departure the duct and dh is the enthalpy difference of the refrigerant through the condenser or the evaporator. Then, the coefficient of performance COP and the cooling capacity QL is calculated from

14 $$\:COP=\frac{{m}_{r}{\left(dh\right)}_{evap}}{SP}=\frac{QL}{SP}$$

where (dh)_evap_ and SP are the enthalpy difference of the refrigerant through the evaporator and the shaft power input to the compressor, respectively. The coefficient of performance for Carnot cycle corresponding to the test rig refrigeration cycle COP_carnot_ is calculated from

15 $$\:CO{P}_{carnot}=\frac{{T}_{L}}{{T}_{H}-{T}_{L}}$$

where T_L_ and T_H_ are the saturated temperature corresponding to the evaporator and condenser pressures. The volumetric efficiency for the swash compressor is calculated from the following equation:

16 $$\:{\eta\:}_{v}=\frac{{m}_{r}}{{\rho\:}_{r}{V}_{d}N}$$

where ρ_r_, V_d_ and N are the density of the refrigerant at entrance the swash compressor, the compressor displacement volume per revolution and compressor shaft rotational speed, respectively. Refrigerant properties are assumed with negligible uncertainty and were taken from tables of F12 presented in^9^. Following the method presented by Moffat^10^, The maximum uncertainties were calculated as: ±0.18 kg/min for mr​ (approx. ±10% of typical values), an absolute uncertainty of **± 0.22 for COP** (representing ± 10.6% at a mid-range COP of 2.07), an absolute uncertainty of ± 0.36 for QL​ (± 9.89%), and an absolute uncertainty of ± 0.08 for ηv​ (± 9.95%). For instance, if the true combined efficiency of the motor and drive system varied between 85% and 95% over the operating range, it would introduce an additional uncertainty of approximately **± 5.5%** in our calculated shaft power (SP). This uncertainty would then propagate directly to the calculated Coefficient of Performance (COP) and volumetric efficiency (ηv​), as both are inversely proportional to SP.

**Table 1:** Specifications of the swash plate compressor used in the present work (SD-508).

Bore	35 mm
Stroke	28.7 mm
Displacement per revolution	138 × 10^3^ mm
Number of cylinders	5

for the experimental test rig.

## Results and discussions

**Fig. 6:**
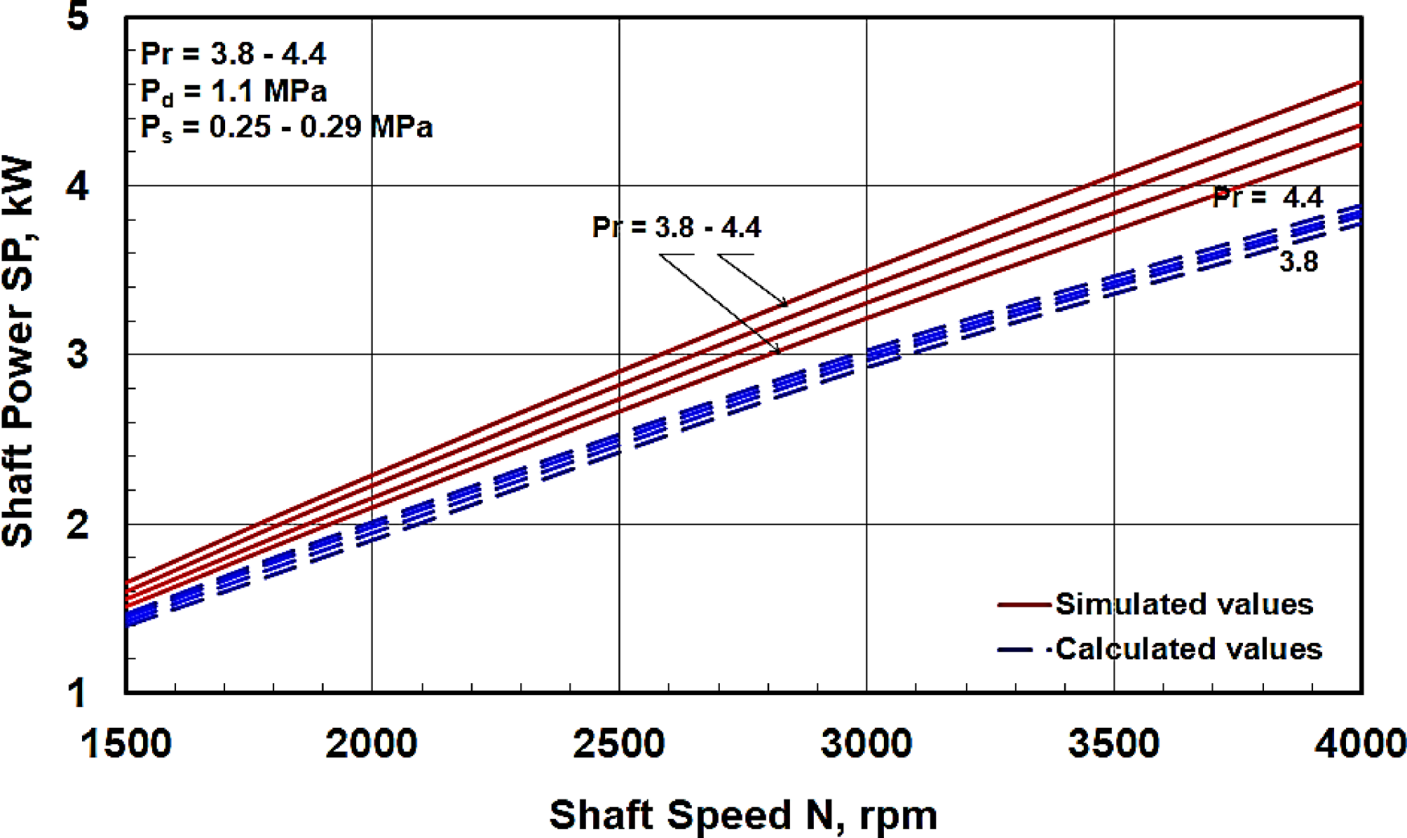
Comparison between the calculated shaft power values with those obtained from the simulated program^8^ at constant P_d_ for different P_s_.

**Fig. 7:**
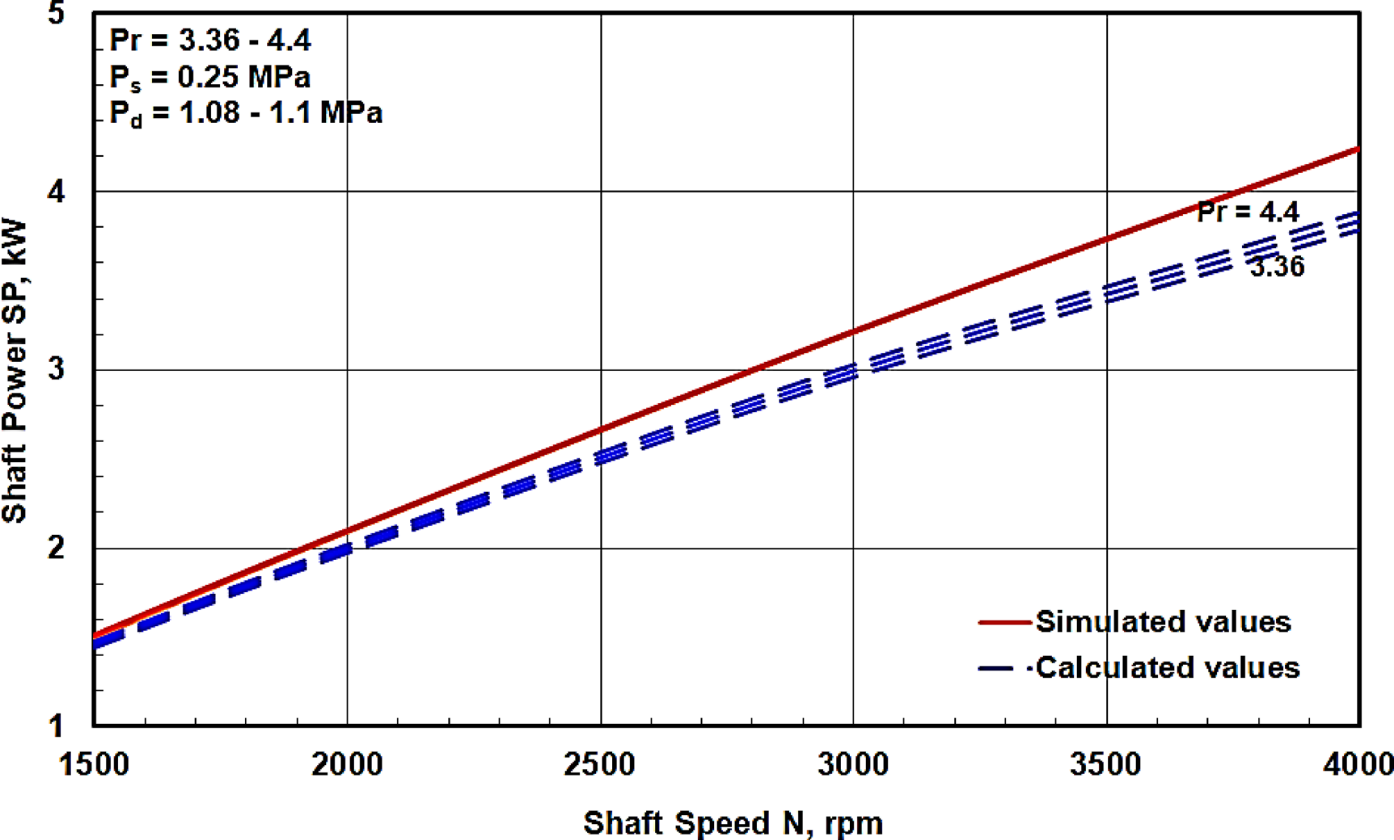
Comparison between the calculated shaft power values with those obtained from the simulated program^8^ at constant P_s_ for different P_d_.

The dimensions of the swash plate compressor employed in the experimental work at which (*r* = 42 mm and α = 18.9^∘^) were used for all theoretical calculations to allow for the reproduction of our results. The calculated values of the minimum shaft power obtained from Eq. (10) were compared with those obtained from the simulation program prepared by the manufacturer^8^. The comparison at a constant discharge pressure Pd for a range of pressure ratios from 3.8 to 4.4 is presented in Fig. 6, and the comparison at a constant suction pressure Ps for a pressure ratio range from 3.36 to 4.4 is shown in Fig. 7. It is evident that the shaft power increases linearly with increasing shaft speed and pressure ratio, which is consistent with the equations derived in the dynamic analysis section.

Figures 6 and 7 reveal that the maximum deviation of the calculated values from the corresponding simulated values is approximately 16% at a shaft speed of 4000 rpm. This discrepancy can be attributed to power losses due to friction between various compressor components, which were neglected in the theoretical analysis. An increase in shaft speed leads to higher frictional power losses, thus contributing to a greater deviation at higher speeds. From Figs. 6 and 7, it can also be observed that the variation in shaft power values due to changes in Ps at a constant Pd is less significant compared to the variation observed due to changes in Pd at a constant Ps. This implies that the shaft power is more influenced by the discharge pressure Pd than by the suction pressure Ps. This observation aligns with Fig. 4, where the discharge pressure impacts a larger duration per shaft revolution. It can be concluded that the current calculations can be utilized to predict the shaft power of a swash plate compressor with acceptable agreement.

**Fig. 8:**
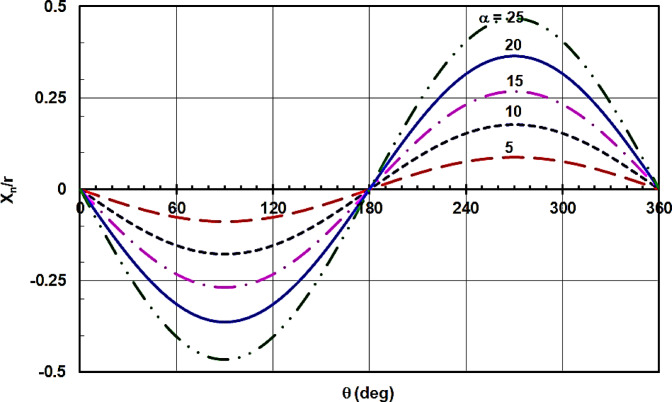
Piston displacement X_n_ relative to r against its angular position θ_n_ for different swash plate inclined angle α.

**Fig. 9:**
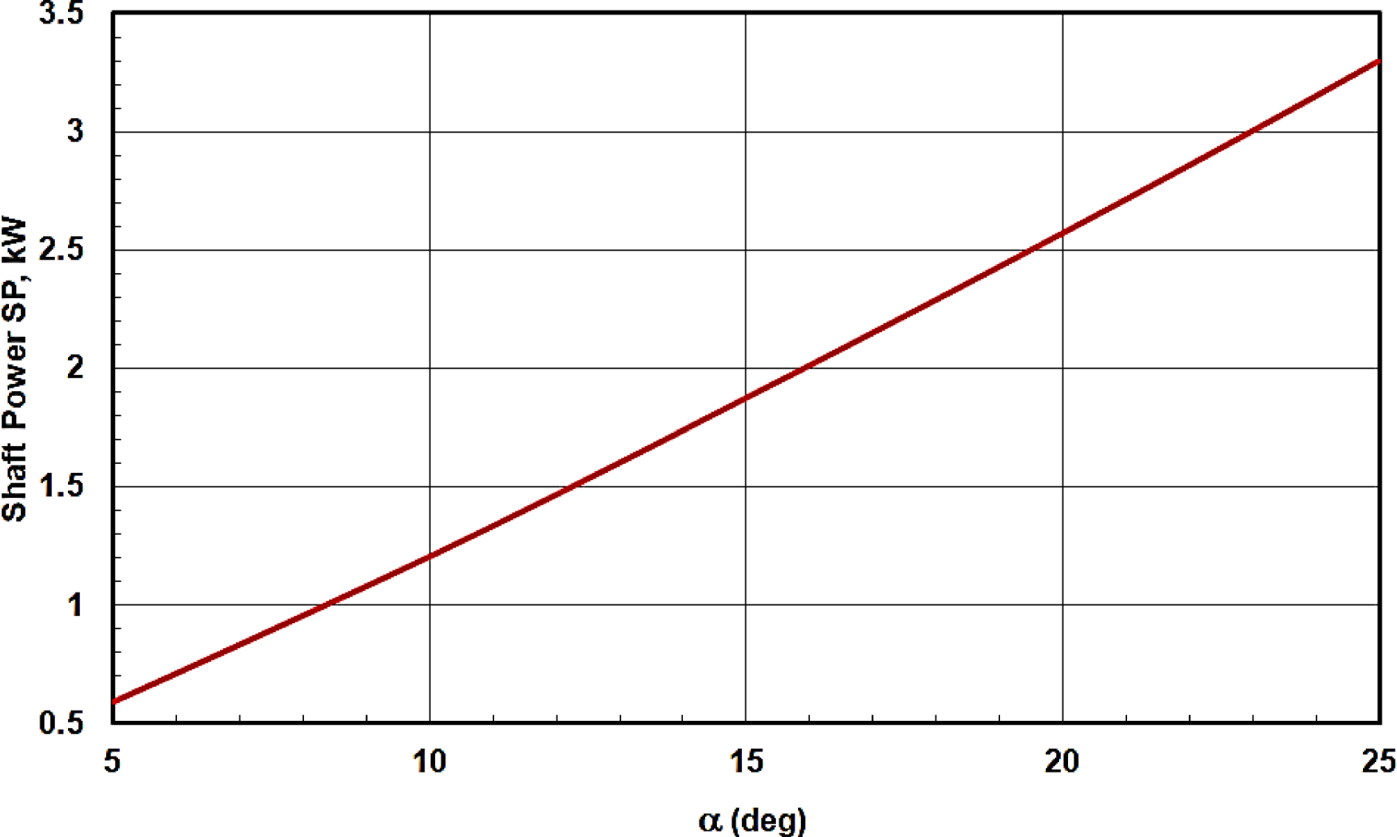
Variation of the shaft power input to the swash plate compressor with the swash angle α.

Figure 8 illustrates the piston displacement X_n_ relative to r versus its angular position θ_n_ for different swash plate inclination angles α. The figure clearly shows that the maximum value of X_n_, which corresponds to the stroke length of the swash plate compressor as indicated in the kinematic analysis, increases with increasing α. Conversely, the shaft power values decrease as α decreases, as is evident from Fig. 9. Therefore, in design scenarios requiring a long stroke and minimal shaft power for the swash plate compressor, the present analysis proves highly valuable for selecting the appropriate swash plate inclination angle and stroke length.

**Fig. 10:**
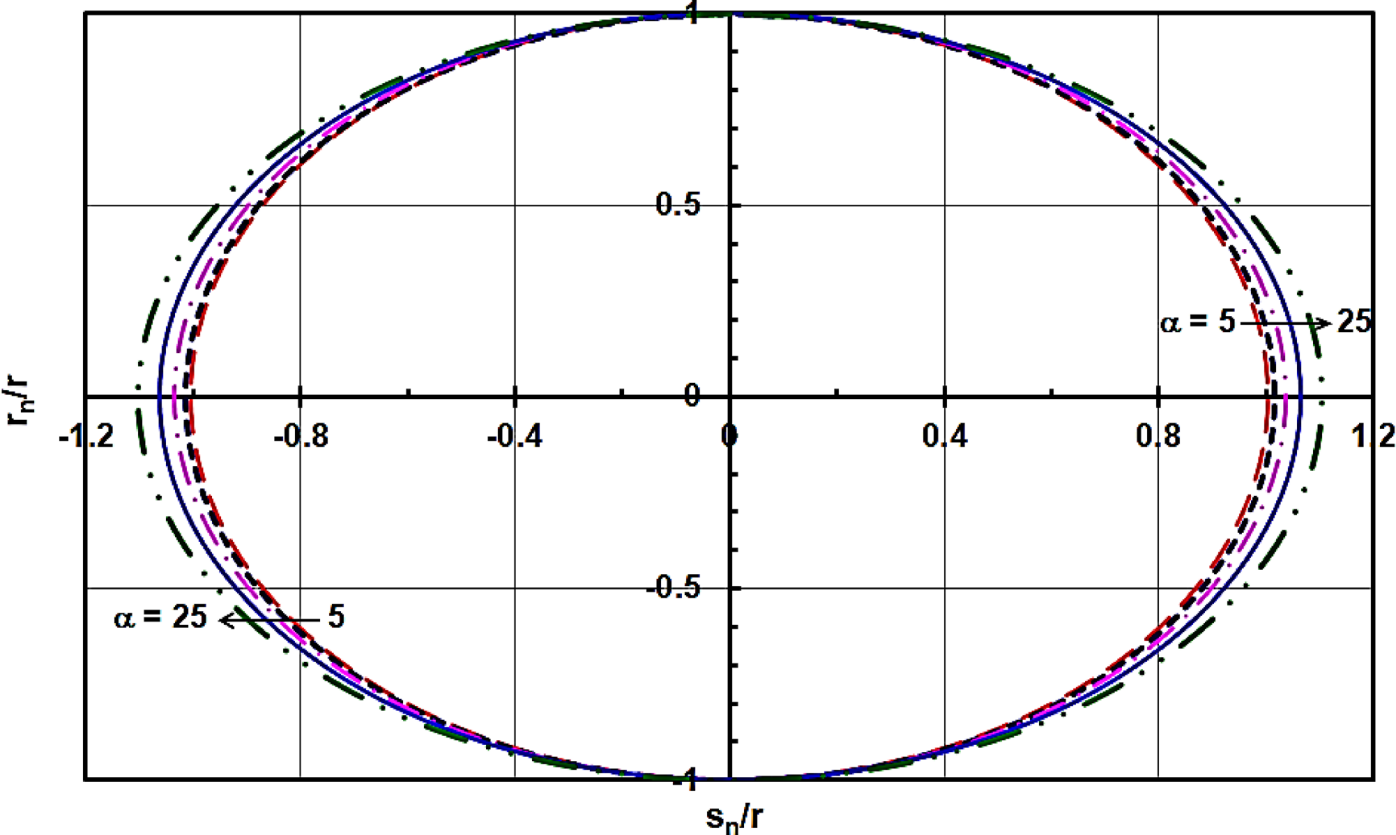
Position of the slipper (r_n_, s_n_) along one revolution for different swash plate inclined angle α.

Figure 10 depicts the slipper position (r_n_, s_n_) over one cycle for various swash plate inclination angles $\alpha$. This figure demonstrates that an increase in α leads to an increase in the value of r_n_, which extends the torque arm, and an increase in the value of α, which lengthens the sliding distance between the slipper and the swash plate. Naturally, an increase in the torque arm necessitates greater shaft power for the swash plate compressor. Additionally, an extended sliding distance results in increased power losses due to friction between the slipper and the swash plate, further contributing to the required shaft power for the swash plate compressor. Consequently, it can be concluded that a small value of α is crucial for minimizing power losses due to friction between the slipper and the swash plate and reducing the overall shaft power required for the swash plate compressor.

**Fig. 11:**
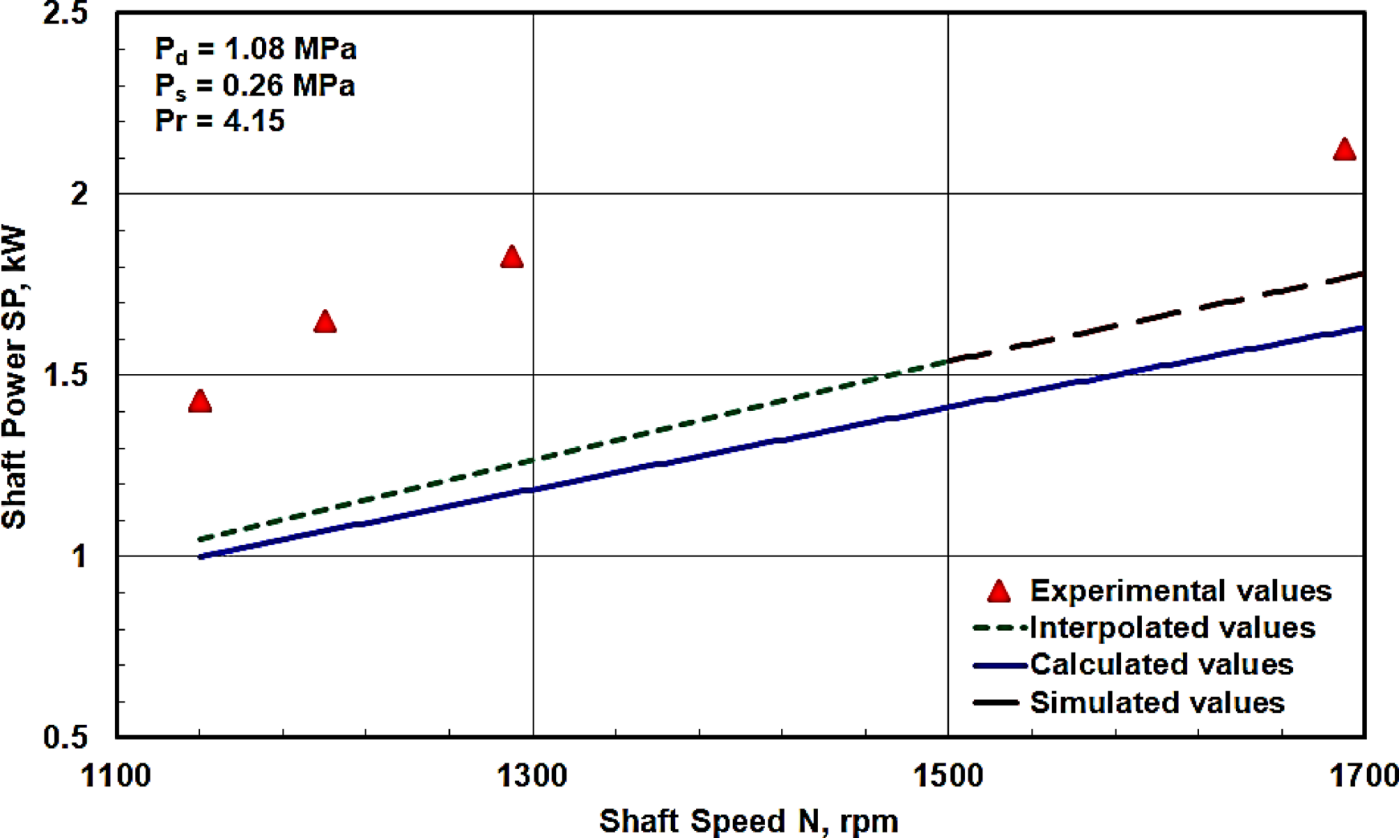
Comparisons between the shaft powers obtained from the calculation, simulation program and the interpolation with the experimental values at various rotational speeds.

The experimental and calculated values of the shaft power for the swash plate compressor are compared with those obtained from the simulation program or from interpolated simulated values prepared by the manufacturer^8^. These comparisons are presented in Fig. 11. These comparisons indicate that at the worst point, the experimental value deviated from the simulated value by approximately 0.6 kW. This discrepancy might be attributed to the use of an older swash plate compressor in experimental testing.

**Fig. 12:**
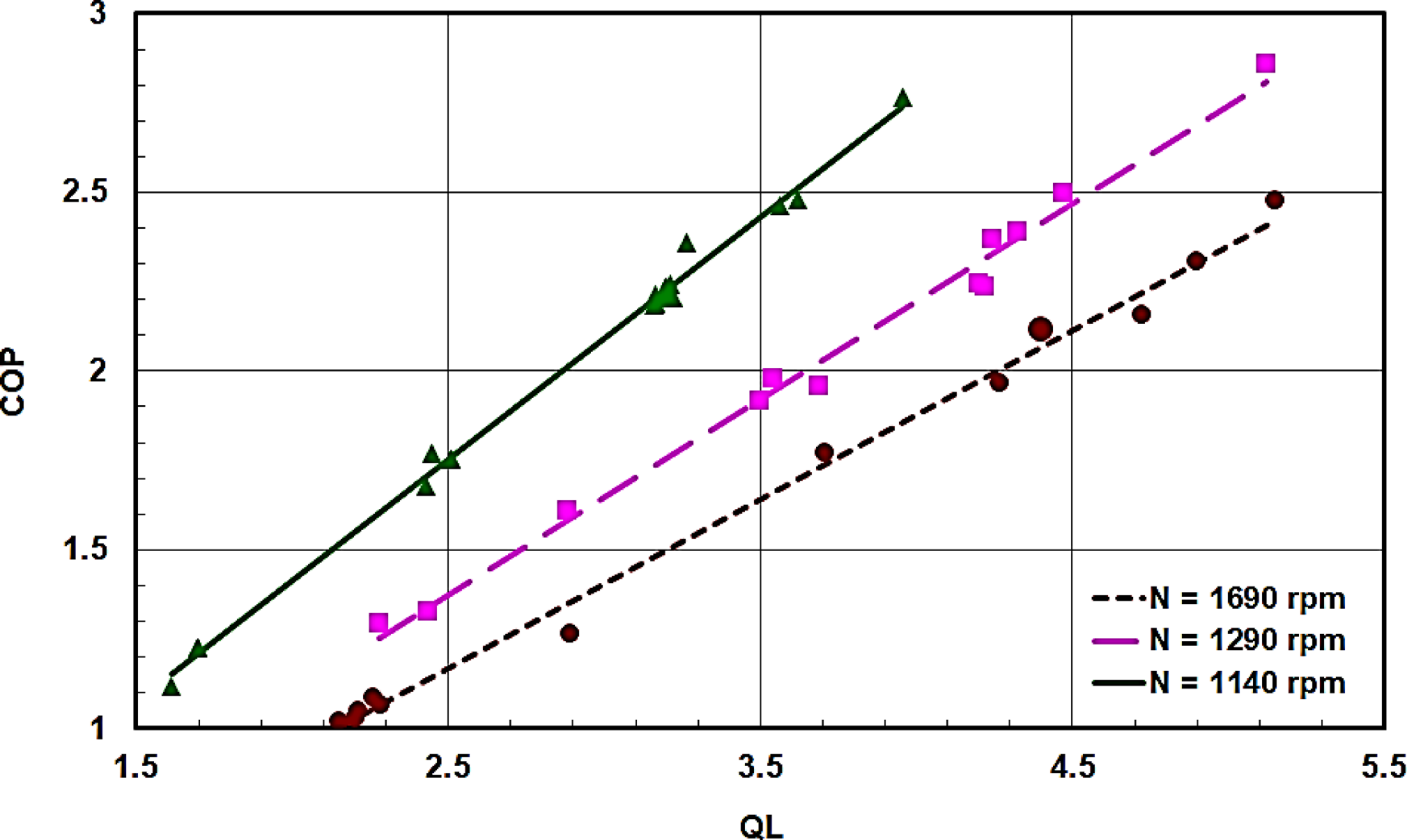
COP against QL at different swash compressor shaft speeds N.

**Fig. 13:**
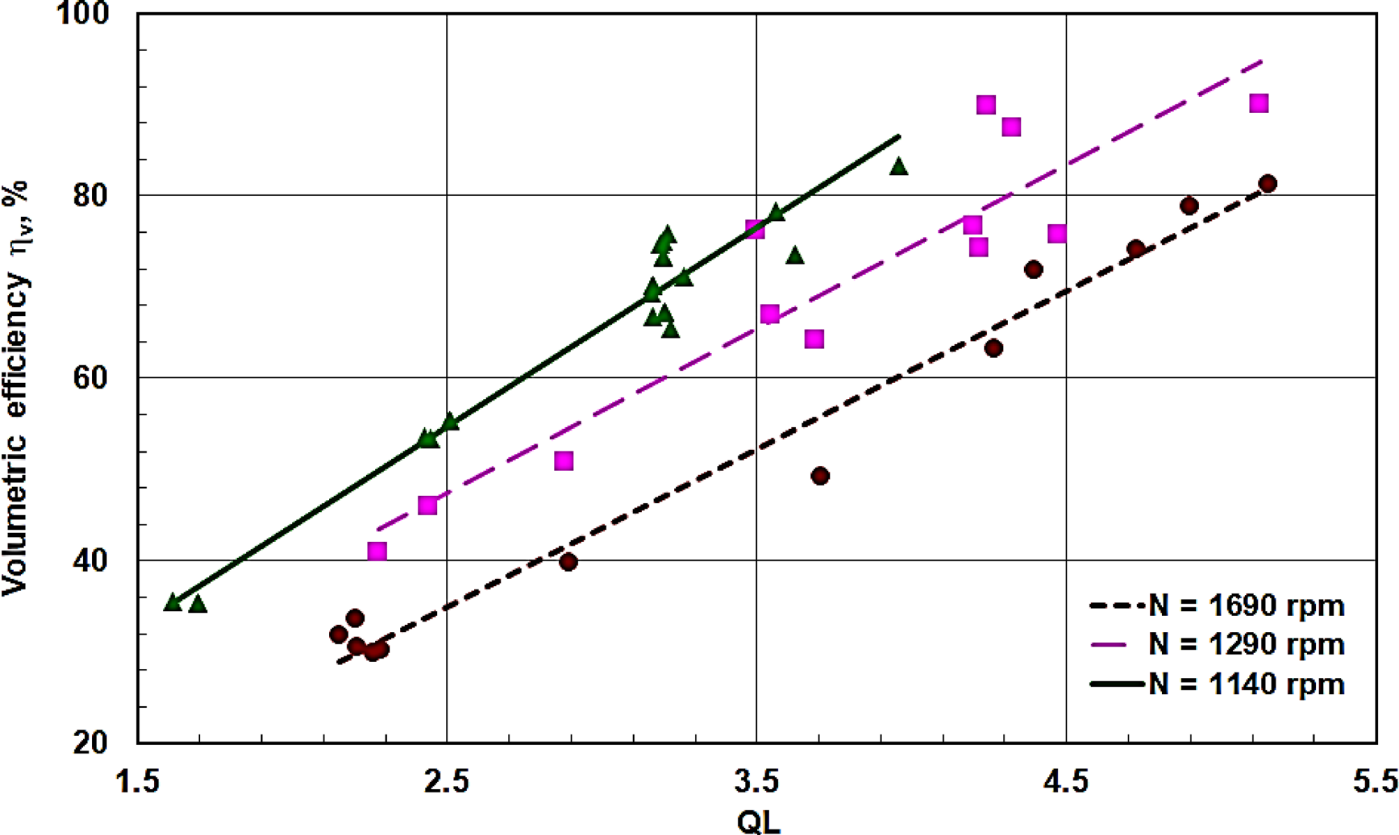
The swash compressor volumetric efficiency η_v_ against QL at different shaft speeds N.

Cooling capacity QL at different swash plate compressor shaft speeds N is plotted against the coefficient of performance COP in Fig. 12, and against the compressor’s volumetric efficiency η_v_ in Fig. 13. It is evident that both COP and η_v_ increase with increasing cooling capacity or increasing shaft speed. This observation aligns with the behavior of conventional vapor compression cycles. Therefore, low rotational speeds and a high value of QL are desirable to achieve high values for both COP and η_v_.

**Fig. 14:**
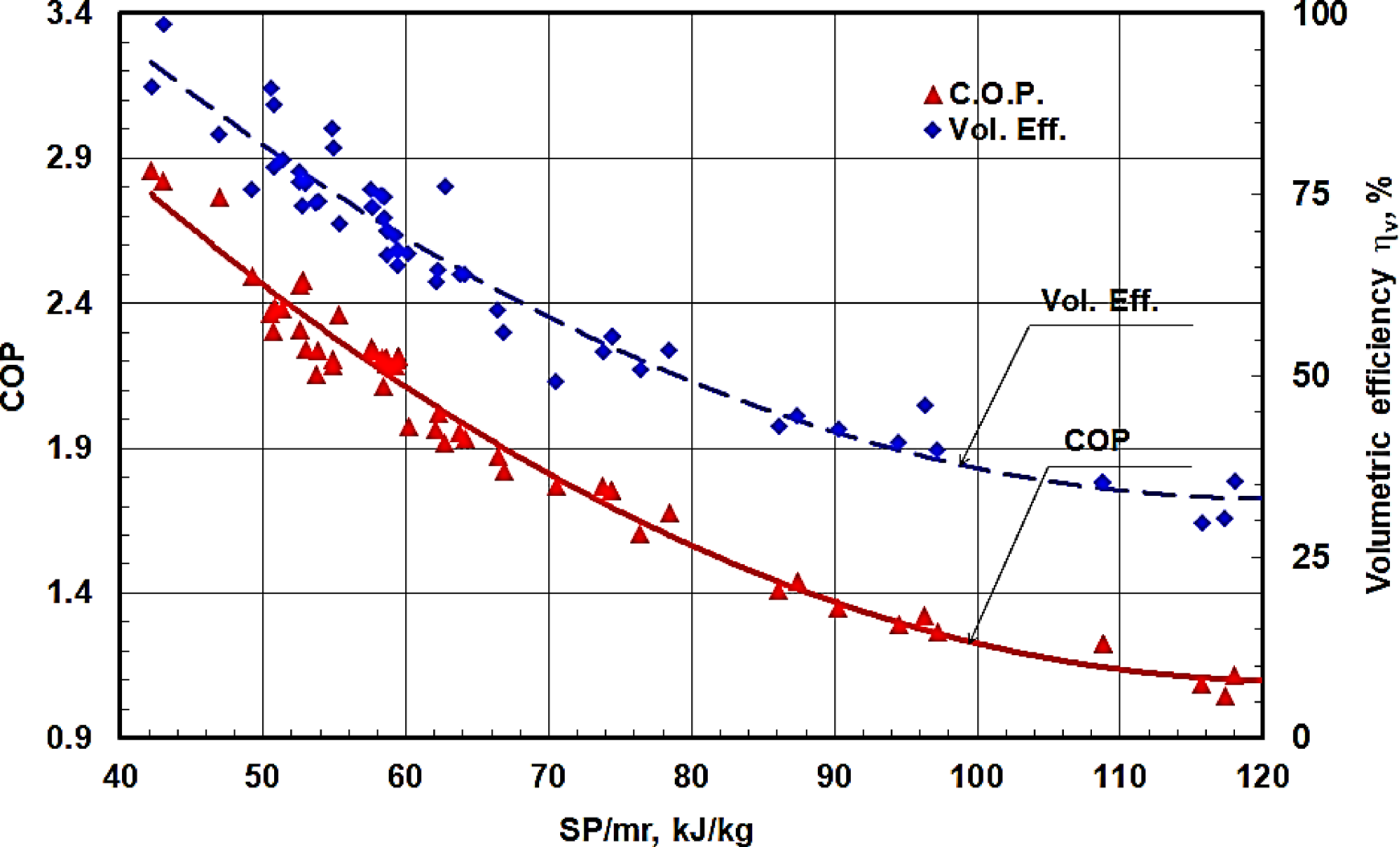
COP and η_v_ against the shaft power per unit refrigerant mass flow rate SP/mr for all the experimental results at various speeds.

**Fig. 15:**
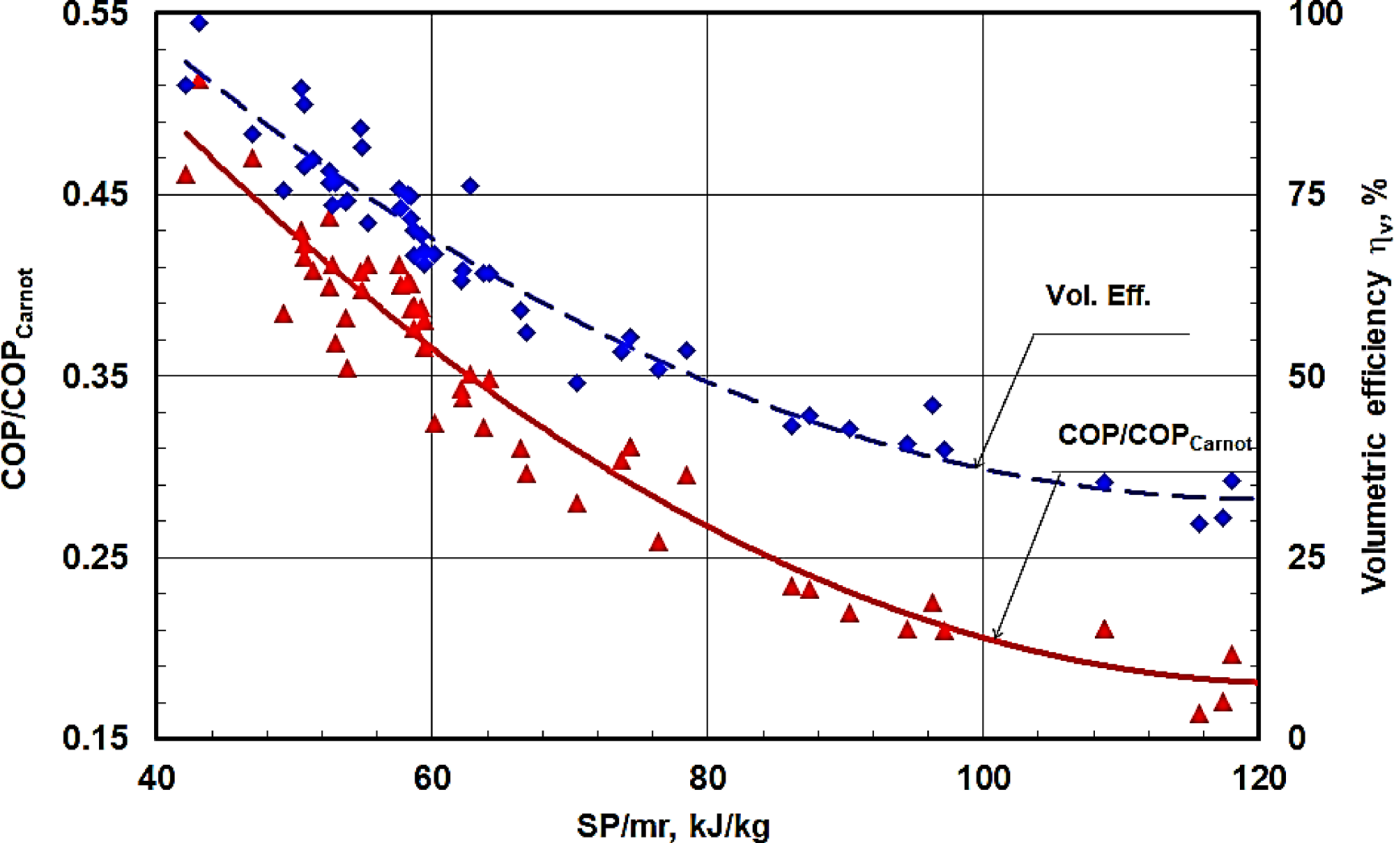
COP/COP_Carnot_ against the shaft power per unit refrigerant mass flow rate SP/mr for all the experimental results at various speeds.

Figure 14 shows that the volumetric efficiency and the coefficient of performance against the shaft power per unit refrigerant mass flow rates for all experimental results at various rotational speeds. It is shown that both the coefficient of performance and the volumetric efficiency is hyperbolically decreased by increasing the shaft power per unit refrigerant mass flow rates. The volumetric efficiency approaches 100% at a low value of the shaft power per unit refrigerant mass flow rate of about 42 kJ/kg. The coefficient of performance relative to that obtained from the corresponding Carnot cycle against the shaft power per unit refrigerant mass flow rate for all experimental results at various rotational speeds is shown in Fig. 15. It is shown that the COP relative to that obtained for the corresponding Carnot cycle is also hyperbolically decreased by increasing the shaft power per unit refrigerant mass flow rate. The maximum value of the COP relative to that obtained for the corresponding Carnot cycle is about 0.46 at SP/mr of 42 kJ/kg. This value is low due to the use of the old compressor.

As it is indicated in the above discussion, while a smaller swash plate angle (α) improves efficiency by reducing frictional losses and shaft power, it also directly reduces the piston stroke (L = 2rtanα). This, in turn, lowers the compressor’s volumetric displacement and overall cooling capacity (QL​). Therefore, the selection of α in compressor design requires balancing the competing objectives of high efficiency (favoring a small α) and high cooling capacity (favoring a large α).

## Conclusions

Based on the theoretical and experimental investigations into the performance of a swash plate compressor integrated into an automotive air conditioning system, the following key conclusions can be drawn:

- The displacements and velocity of the swash plate mechanism can be accurately described as functions of the compressor’s geometry and angular speed.
- The average values of shaft torque and shaft power for the swash plate compressor can be precisely represented as functions of the piston’s angular position, the swash plate inclination angle, the eccentricity of the piston’s center from the swash plate’s center, the pressure-holding angle, and the compressor’s suction and discharge pressures.
- The analytical equations developed in this study provide a reliable tool for predicting the shaft power of a swash plate compressor with acceptable accuracy.
- The maximum piston displacement, which corresponds to the stroke length of the swash plate compressor, increases directly with an increase in the swash plate inclination angle. Conversely, the shaft power values decrease as the swash plate inclination angle decreases. This finding is particularly significant for compressor design, highlighting that in scenarios demanding a long stroke with minimal shaft power, the current analysis is crucial for selecting the optimal swash plate angle and stroke length.
- A small swash plate inclination angle is essential for minimizing power losses attributed to friction between the slipper and the swash plate, consequently reducing the overall shaft power required for the swash plate compressor.
- Shaft power exhibits a linear increase with both increasing shaft speed and increasing pressure ratio.
- The variation in shaft power values caused by changes in suction pressure Ps at a constant discharge pressure Pd is less pronounced compared to the variation observed due to changes in Pd at a constant Ps. This indicates that the shaft power is more significantly influenced by the discharge pressure Pd than by the suction pressure Ps.
- Both the coefficient of performance (COP) and the compressor’s volumetric efficiency ηv increase with an increase in cooling capacity, an increase in shaft speed, or a decrease in shaft power per unit mass flow rate of refrigerant. Therefore, achieving high values for both COP and ηv necessitates low rotational speeds, large cooling capacities, and a reduced shaft power per unit mass flow rate of refrigerant.
- The coefficient of performance (COP) relative to the corresponding Carnot cycle exhibits a hyperbolic inverse relationship, decreasing as the shaft power per unit mass flow rate of refrigerant increases.
- The study indications that while a smaller swash plate angle (α) improves efficiency by reducing frictional losses and shaft power, it also directly reduces the piston stroke (L = 2rtanα). This, in turn, lowers the compressor’s volumetric displacement and overall cooling capacity (QL​). Therefore, the selection of α in compressor design requires balancing the competing objectives of high efficiency (favoring a small α) and high cooling capacity (favoring a large α). While we do not have the data to generate a multi-objective map for this paper, it can be suggested as a valuable direction for future design optimization studies.

## Data Availability

the datasets generated and analyzed during the current study are available from the corresponding author upon reasonable request.

## List of symbols

A_P_: Cross sectional area of a single piston, m^2^
COP: Coefficient of performance for the system
COP_Carnot_: Coefficient of performance for Carnot cycle corresponding to the system
Cp_a_: Air specific heat at duct entrance, kJ/kg.K
Dh: Enthalpy difference in the refrigerant through the condenser or the evaporator
m_a_: Mass flow rate of air flowing through duct, kg/min
m_P_: Mass of single piston, kg
m_r_: Refrigerant mass flow rate, kg/min
N: Number of the pistons
N: Piston number
N: Rotational speed of the Compressor shaft, rpm
Np: The number of the pistons
P_a_: Pressure at air duct entrance, kPa
P_d_: Discharge pressure, kPa
P_n_: Pressure acting on the n^th^ piston, kPa
P_s_: Suction pressure, kPa
QL: Cooling capacity, kW
R: Gas constant for air, kJ/kg.K
R: Offset of the piston center from the swash plate center, m
r_n_: Position of the slipper on the swash plate along Y axis
R_n_: Swash plate force acting on the piston, kN
s_n_: position of the slipper on the swash plate plane in normal direction to Y axis
SP: Compressor shaft power, kW
T: Net instantaneous torque, kN.m
T_a_: Average temperature at air duct entrance, K
T_av_: Average torque exerted on the driven shaft
T_H_: Saturated temperature corresponding to the condenser pressure, K
T_L_: Saturated temperature corresponding to the evaporator pressure, K
V_a_: Air velocities through air duct, m/s
X_n_: the displacement of the n^th^ piston, m
$\:{\ddot{X}}_{n}$: Instantaneous translational acceleration of the n^th^ piston

## **Greek Symbols**

$\alpha$: Swash plate inclined angle
$\Delta\:{\rm T_{a}}$: Difference between the average temp. of the air at entrance and departure the duct, K
$\gamma$: Pressure carry over angle
$$\eta\:_{\rm v}$$: volumetric efficiency
${\rm \theta_1}$: Position angle of the first piston
${\rm \theta_n}$: Angular position of the n^th^ piston
${\rm \rho\:r}$: Refrigerant density, kg/m^3^
$\omega$: Angular velocity of the swash plate
